# The role of viral evolution in rabies host shifts and emergence

**DOI:** 10.1016/j.coviro.2014.07.004

**Published:** 2014-10

**Authors:** Nardus Mollentze, Roman Biek, Daniel G Streicker

**Affiliations:** 1 Institute of Biodiversity, Animal Health and Comparative Medicine, University of Glasgow, Glasgow G12 8QQ, UK; 2 Medical Research Council, University of Glasgow, Centre for Virus Research, Glasgow G61 1QH, UK

## Abstract

Despite its ability to infect all mammals, *Rabies virus* persists in numerous species-specific cycles that rarely sustain transmission in alternative species. The determinants of these species-associations and the adaptive significance of genetic divergence between host-associated viruses are poorly understood. One explanation is that epidemiological separation between reservoirs causes neutral genetic differentiation. Indeed, recent studies attributed host shifts to ecological factors and selection of ‘preadapted’ viral variants from the existing viral community. However, phenotypic differences between isolates and broad scale comparative and molecular evolutionary analyses indicate multiple barriers that *Rabies virus* must overcome through adaptation. This review assesses various lines of evidence and proposes a synthetic hypothesis for the respective roles of ecology and evolution in *Rabies virus* host shifts.

## Highlights

• *Rabies virus* has a broad host range, but transmission cycles are species-specific.
• Significant barriers limit viral establishment in new host species, but are poorly characterised.
• Viral preadaptation may facilitate emergence, but does not rule out host adaptation.
• Several lines of evidence point to adaptation of *Rabies virus* to specific hosts.

**Current Opinion in Virology** 2014, **8**:68–72

This review comes from a themed issue on **Virus evolution**

Edited by **Michael Brockhurst** and **Scott Hensley**

For a complete overview see the Issue and the Editorial

Available online 26th July 2014

**http://dx.doi.org/10.1016/j.coviro.2014.07.004**

1879-6257/© 2014 The Authors. Published by Elsevier Ltd. This is an open access article under the CC BY license (http://creativecommons.org/licenses/by/3.0/).

## Introduction

*Rabies virus* (RV) is a notorious multi-host pathogen that is capable of infecting all mammals, but paradoxically is maintained in distinct host species-associated transmission cycles, typically within the Carnivora and Chiroptera [1]. However, not all carnivores and bats are reservoirs, and potential reservoirs outside these orders occasionally appear, such as the apparent transmission of rabies amongst greater kudu antelope in Namibia and among non-human primates in Brazil [2, 3]. The factors that contribute to reservoir capacity, limit onward transmission by incidentally infected species and prevent variants associated with one host species from transmitting in another host species are poorly understood.

**Figure 1:**
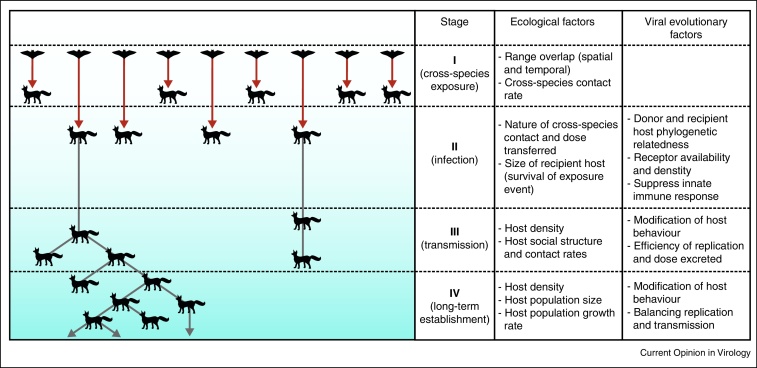
The ecological and evolutionary factors affecting *Rabies virus* host shifts at various stages. Despite many cross-species transmission events, and presumably even more unsuccessful exposures, comparatively few infections result in onward spread in the recipient species. An even smaller number of such outbreaks result in long term establishment of RV in the new host species. In the simplest case, the cross-species emergence of RV is determined purely by ecological factors (here termed the ‘ecology only’ model). However, several lines of evidence point to a need for viral adaptation to the recipient host to allow progression to the next stage.

The host-species association of RV is thought to arise from rare historical jumps into new species, which are followed by predominately within-species transmission [4]. Such cross-species emergence proceeds in various stages — after exposure (stage I, Figure 1), the pathogen must be able to infect the novel recipient host (stage II), this single infection must result in onward transmission to con-specifics (stage III), and such transmission must be maintained (stage IV). Contemporary cross-species emergence events are of concern in conservation, and — by increasing the risk of human exposures — in public health. Recent examples include the emergence of domestic dog-associated RV in endangered Ethiopian wolves, of big brown bat-associated RV in striped skunks and gray foxes, and of striped skunk-associated RV in gray foxes [5, 6•, 7••]. Yet, in none of these examples did RV establish permanent transmission cycles in the new host species. While human intervention has contributed to this, a key question then is to what extent adaptive evolution is required for RV to cross species barriers — understanding this could help to anticipate which host shifts are most likely to occur and where new reservoirs may emerge.

The simplest hypothesis is that RV host shifts are determined purely by the ecological factors that provide opportunities for cross-species transmission and that genetic differences between host-associated variants arise from neutral evolutionary processes (here termed the ‘ecology only’ model, Figure 1). Indeed, some RV outbreaks in novel host species have been associated with little to no genetic change [6•, 7••], and the RV genome is known to be subject to strong purifying selection [8]. Here, in reviewing lines of evidence from the distribution of RV reservoirs among mammals, comparative studies, *in vivo* and *in vitro* experiments, and recent rabies outbreaks resulting from cross-species transmission events, we assess whether ecological opportunity alone is necessary and sufficient for RV host shifts. We argue that broad scale historical patterns of cross-species transmission and host shifts, molecular evolutionary analyses of successful host shifts and phenotypic differences among host-associated RV variants all suggest a role for adaptive evolution in the establishment of new rabies reservoirs. We further propose a synthetic hypothesis for the respective roles of ecology and evolution in RV host shifts.

## Non-evolutionary barriers to *Rabies virus* host shifts

Intrinsic features of the species involved may prevent some cross-species exposures from producing infection (stage II, Figure 1) in all but exceptional circumstances. For example, thick hides may prevent inoculation during a bite and small body size may reduce the likelihood of surviving an encounter with a larger, rabid animal. After exposure, the density of nicotinic acetylcholine receptors for viral cell entry into host cells may influence the permissiveness of different host species to productive viral infection [9]. Furthermore, many mammalian groups, including rodents, primates and ungulates are commonly infected by other host species, but rarely maintain RV independently [10, 11, 12]. This points to the possibility of further intrinsic physiological or ecological barriers to establishment of RV reservoir hosts that may be impossible to overcome through viral evolution. For example, dental structures that are unlikely to pierce the skin of con-specifics will limit chains of transmission (stage III, Figure 1). Ecological factors such as low population densities or lack of aggregations of individuals, may similarly prevent onward transmission by reducing intra-specific contact rates.

## Macro-evolutionary and micro-evolutionary patterns in *Rabies virus* host shifts

Among species that are commonly infected, the ‘ecology only’ model of RV host shifts would predict (i) variation in the degree of host-association among viral lineages due to variation in inter-specific contact rates among different reservoir hosts and (ii) that spill-over transmission and host shifts should be correlated with ecological overlap rather than the phylogenetic relatedness of host species. These predictions have been testable using large-scale phylogenetic analyses across many host species. Although certain reservoirs seem predisposed to infecting other species, true multi-host RVs (i.e. those that are maintained by multiple host species [13]) remain conspicuously absent [14, 15, 16]. The second prediction on the relative roles of ecological overlap and host relatedness has been tested for RVs in North American bats. There, both initial cross-species transmission (stage II, Figure 1) and the likelihood of establishment in novel hosts (stages III and IV) were more closely associated with host phylogenetic relatedness than the extent of ecological overlap between species, perhaps because related hosts require less viral adaptation [16, 17]. Interestingly, a recent comparative analysis across 34 host species also demonstrated non-random clustering of RV reservoirs on the carnivore phylogeny, highlighting the potential for host phylogeny to constrain the diversity of RV reservoirs [18^•^].

At the molecular level, evidence for positive selection in functional regions following host shift events would be suggestive of a role for adaptive evolution in RV host shifts. The nucleoprotein-encoding and glycoprotein-encoding genes (N and G genes, respectively) of RV isolates from various hosts are typically subject to strong purifying selection, with evidence for positive selection limited to a few G sites [4, 8, 19]. However, these analyses used computational techniques that assumed pervasive positive selection across the entire RV phylogeny — unlikely the case for a virus experiencing distinct host environments. A more recent analysis instead found evidence for episodic bouts of positive selection on many sites across the G and polymerase (L) genes that appeared to be associated with host shifts among bats [20^••^]. This analysis also revealed distinct viral evolutionary pathways during adaptation to each bat species that may have depended on the genotype of the introduced virus. Thus, the balance of pre-adapted genetic variation and post-emergence evolution could shape the overall likelihood of a host shift. From these studies, a picture emerges of altered selection pressures immediately following host shifts leading to adaptive changes whose extent and genomic distribution may depend on the specific viral variant involved. Subsequent purifying selection presumably reflects the advantageous nature of adaptive changes in the novel host, constraints from the need to replicate in multiple cell types and the absence of strong immunological pressure [8].

## Insights from cell culture and *in vivo* infection studies

Controlled experiments in which RVs are inoculated into atypical host species or cell lines offer further insights into the role of adaptive evolution in host shifts. Such studies have repeatedly demonstrated phenotypic differences among viral variants that may reflect optimisations to host ecological factors such as contact-rates to ensure sustained transmission (stage III and IV, Figure 1; [21, 22]). For example, bat-associated RVs show a general increase in incubation and morbidity period in both mice and carnivores with decreasing gregariousness of the bat host [21]. Differences in tissue tropism and cell entry, particularly at low temperatures [22, 23] and a correlation between the neutral evolutionary rates of bat RVs and their hosts’ seasonal activity patterns [24] further point to variable persistence strategies across host species. Thus, RV may need to adapt to decreased (or increased) opportunities for transmission depending on the life history and behaviour of its host species. Such ‘fine-tuning’ of disease progression has been demonstrated in red foxes, where the timing from the death of the first host to death of the second was significantly less variable in a modern fox-associated isolate compared to one collected 10 years prior [25]. At the molecular level, viral evolution within new host species may be facilitated by ample sub-consensus genetic variation on which positive selection may act [26].

If host adaptation is indeed the explanation for phenotypic differences among RV variants, one may expect a decreased ability to infect other species or patterns of clinical disease in incidental hosts that are less likely to lead to onward transmission. Although limited by low sample sizes, several heterologous host infection studies appear to demonstrate just that. When raccoons were inoculated with a raccoon-associated isolate or a dog-associated isolate, the homologous strain caused a long incubation period leading to acute clinical signs, whereas the heterologous dog strain caused only subtle neurological signs such as lethargy [27]. These differences appeared to be linked to the regions of the brain successfully infected [27]. Decreased ability to infect heterologous species was also demonstrated using RVs from striped skunks, which caused lethal infection in their natural hosts but failed to infect raccoons, despite both species being known reservoirs of RV in nature [28, 29]. Importantly, this appears to be an effect of RV strain, not differences in resistance between the hosts: inoculation of fox-associated RV into striped skunks at a dose 10 times higher than what was sufficient to kill 7/7 foxes failed to kill 6/6 skunks [30]. These differences indicate a clear effect of virus genotype on the predisposition for cross-species transmission. Moreover, Sikes [30] found that the virus titres excreted in fox saliva were lower than the dose required to infect skunks, while the titres generally found in the saliva of experimentally infected skunks were high enough to kill foxes, suggesting a mechanism through which viral genotype effects could influence onward transmission. Interestingly, similar experiments in yellow mongooses revealed no such dose response, despite differences in infectivity among RV strains [31], suggesting an entirely different host barrier.

## Evolutionary change during emergence

The effects of viral genotype on predisposition to host shifts are further illustrated through naturally occurring cross-species emergence events where detailed epidemiological surveillance has been coupled with viral sequencing. Across a series of outbreaks in striped skunks and gray foxes, Kuzmin *et al.* [6^•^] found no evidence of positive selection in the new host species, despite sustained transmission within each species. The outbreaks did however arise from the same bat-associated RV lineage, and there were signs of convergent evolution between this lineage and several carnivore-associated lineages [6^•^]. Such ‘pre-adaptation’ may explain the frequency of host shifts of this lineage to carnivores. It is important to note however that five of the six changes are also present in some other bat lineages [6^•^], suggesting either that minor evolutionary changes in a variety of bat viruses could create viruses capable of onward transmission in carnivores, or that these changes have little to do with the ability to emerge in carnivores.

Another apparent example of pre-adaptation comes from a suspected host shift of skunk-associated RV into gray foxes. Borucki *et al*. [7^••^] showed that this outbreak involved the selection of a rare variant already present below the consensus level in historic samples from the same area. Thus, if rare variants can at least occasionally survive the cross-species transmission bottleneck, they might be further favoured by selection in the recipient host. Indeed, unusually high numbers of fox cases, presumably due to repeated cross-species infections (stage II, Figure 1), were reported in the immediate area of the eventual host shift for several years prior to the event [7^••^]. This seems to indicate that the lineage was already more capable of infecting foxes than other skunk RVs. However, the interpretation of such emergence events is not straightforward. In both of the above cases, onward transmission within the novel species eventually ceased, either because of interventions aimed at halting the outbreaks or due to stochastic factors. Thus it remains unclear whether adaptation within the new host would have occurred as each virus became established or whether the absence of such adaptation ultimately contributed to viral extinction. Similar analyses of successful host shifts in carnivores would be of great utility to determine whether both pre-emergence and post-emergence adaptation are involved in RV host shifts, as suggested by the study of RV jumps among bat species [20^••^].

## Synthesis and future directions

Taken together, all lines of evidence indicate that ecology alone is insufficient to explain the patterns of host shifts observed in RV. Although pre-adaptation may make some RV variants more likely to cross the species barrier than others, phenotypic differences and evidence of selection within recipient species suggest that this is not always sufficient. Once adaptation has occurred, it is maintained by ecological patterns of host-species associations, which provide ample opportunities for within-species transmission but far fewer opportunities for cross-species transmission. This ecological isolation sets the stage for purifying selection to maintain adaptive changes. This course of events may have great consequences on viral emergence. More reservoirs would enable exploration of more genomic space, increasing the likelihood of rare pre-adapted variants arising and potentially leading to a ‘snowball effect’ of ever increasing viral emergence. The importance of both pre-adaptation and post-emergence evolution may also explain the apparent paradox of constraints on host shifts, even to permissible hosts, despite the vast potential for rapid genetic change in RNA viruses. It could be that many potential host shifts were doomed from the beginning because of an inappropriate starting virus (Figure 1).

Many questions regarding the adaptation of RV to hosts remain unanswered. For example, there is still no clear picture of the determinants of effective RV reservoirs. In this regard, a meta-analysis of the traits associated with known reservoirs may shed some light. The taxonomic scale of the effect of host phylogeny on emergence and establishment, the ecological or physiological mechanisms that constitute this barrier and the viral genotypic and phenotypic changes that overcome it also remain unknown. Finally, the seeming emergence of new, non-traditional reservoir species such as coatis [32], kinkajous [33] and marmosets [3] raises questions on whether this is a real phenomenon or the effects of improving surveillance and genetic typing methods. The fact that these novel RV lineages often come from historically unobserved areas points to the latter explanation, but it still remains unclear whether these viruses persist independently in the sampled animals or some other reservoir. Such questions could be answerable with increased surveillance and field studies in non-traditional host species and phylogenetic reconstruction of the most recent common ancestors of these variants.

If we hope to understand RV adaptation, there is a clear need for full-genome sequencing studies, and perhaps even deep sequencing, to allow assessment of the roles of standing diversity and selection within donor and recipient hosts. Questions also remain regarding the repeatability of RV host shifts [20^••^], which can best be answered using replicated *in vitro* or *in vivo* experiments. Synthesising ecological, virological and genomic studies provides a promising way forward to anticipate the fate of future RV emergence events and identify prospective new reservoir species.

## References and recommended reading

Papers of particular interest, published within the period of review, have been highlighted as:

• of special interest
•• of outstanding interest
